# Association of sub-acute changes in plasma amino acid levels with long-term brain pathologies in a rat model of moderate-severe traumatic brain injury

**DOI:** 10.3389/fnins.2022.1014081

**Published:** 2023-01-06

**Authors:** Xuan Vinh To, Abdalla Z. Mohamed, Paul Cumming, Fatima A. Nasrallah

**Affiliations:** ^1^The Queensland Brain Institute, The University of Queensland, Saint Lucia, QLD, Australia; ^2^Thompson Institute, University of the Sunshine Coast, Sunshine Coast, QLD, Australia; ^3^Department of Nuclear Medicine, Bern University Hospital, Bern, Switzerland; ^4^School of Psychology and Counselling, Queensland University of Technology, Brisbane, QLD, Australia; ^5^Centre for Advanced Imaging, The University of Queensland, Saint Lucia, QLD, Australia

**Keywords:** traumatic brain injury, brain microstructure, amino acids, diffusion magnetic resonance imaging, neurite orientation dispersion and density imaging (NODDI)

## Abstract

**Introduction:**

Traumatic brain injury (TBI) induces a cascade of cellular alterations that are responsible for evolving secondary brain injuries. Changes in brain structure and function after TBI may occur in concert with dysbiosis and altered amino acid fermentation in the gut. Therefore, we hypothesized that subacute plasma amino acid levels could predict long-term microstructural outcomes as quantified using neurite orientation dispersion and density imaging (NODDI).

**Methods:**

Fourteen 8–10-week-old male rats were randomly assigned either to sham (*n* = 6) or a single moderate-severe TBI (*n* = 8) procedure targeting the primary somatosensory cortex. Venous blood samples were collected at days one, three, seven, and 60 post-procedure and NODDI imaging were carried out at day 60. Principal Component Regression analysis was used to identify time dependent plasma amino acid concentrations after in the subacute phase post-injury that predicted NODDI metric outcomes at day 60.

**Results:**

The TBI group had significantly increased plasma levels of glutamine, arginine, alanine, proline, tyrosine, valine, isoleucine, leucine, and phenylalanine at days three-seven post-injury. Higher levels of several neuroprotective amino acids, especially the branched-chain amino acids (valine, isoleucine, leucine) and phenylalanine, as well as serine, arginine, and asparagine at days three-seven post-injury were also associated with lower isotropic diffusion volume fraction measures in the ventricles and thus lesser ventricular dilation at day 60.

**Discussion:**

In the first such study, we examined the relationship between the long-term post-TBI microstructural outcomes across whole brain and the subacute changes in plasma amino acid concentrations. At days three to seven post-injury, we observed that increased plasma levels of several amino acids, particularly the branched-chain amino acids and phenylalanine, were associated with lesser degrees of ventriculomegaly and hydrocephalus TBI neuropathology at day 60 post-injury. The results imply that altered amino acid fermentation in the gut may mediate neuroprotection in the aftermath of TBI.

## Introduction

Traumatic brain injury (TBI) and its sequelae are a major public health issue and a leading cause of mortality and disability worldwide, with an estimated global annual incidence of 295 per 100,000 population (Nguyen et al., 2016). Effects of TBI on human brain structure and function are inherently complex, with a broad range of causative mechanisms, injury severity, and clinically diverse presentations (William and Rajajee, 2022). Part of this complexity stems from the distinction between the direct effects such as contusion, hematoma, axonal integrity (Alves and Marshall, 2006), and the secondary brain injury, which manifests in edema, increased intracranial pressure, mitochondrial and metabolic dysfunction, excitotoxicity, oxidative stress, vascular injury, neuroinflammation, perfusion changes, even extending to apoptosis and necrosis (Kaur and Sharma, 2017). A wide range of signaling pathways contribute to these secondary processes, including ions such as Ca^2+^, K^+^, and Na^+^, and also signaling molecules such as adenosine triphosphate (ATP), neurotransmitters and their receptors, as well as reactive oxygen species, amino acids, immune cytokines and chemokines, and apoptosis regulators (Kaur and Sharma, 2017). Blood levels of many of these markers present themselves as prognostic indicators of TBI outcome, notably neuroinflammation modulators like tumor necrosis factor alpha (TNFα), interleukins (Woodcock and Morganti-Kossmann, 2013), S100 astroglial calcium-binding protein beta (S100β), glial fibrillary acidic protein (GFAP), neuronal specific enolase (NSE), and ubiquitin C-terminal hydrolase-L1 (UCH-L1) (Vos et al., 2004; Chabok et al., 2012; Mercier et al., 2013; Zetterberg and Blennow, 2016).

Amino acids are the building blocks of proteins, which in their diverse forms, are key structural and functional constituents of living cells (Dietzen, 2017). In the brain, amino acids are involved in neurotransmission, and more generally in the development, maintenance, repair, and recovery of neural tissues; changes in the brain amino acid pools can contribute to central nervous system pathologies (Kurbat and Lelevich, 2009). Acute TBI can result in changes in the gut microbiota, which may entail alterations in amino acid fermentation (Celorrio and Friess, 2022). We therefore supposed that blood concentrations of amino acids might bear some relation to the progression of TBI, thus presenting a potential diagnostic or prognostic tool. Indeed, several prior studies have examined changes in plasma or serum amino acid levels after TBI in humans (Deutschman, 1987; Flakoll et al., 1995; Petersen et al., 1996; Vuille-Dit-Bille et al., 2012; Jeter et al., 2013) and in animal models (Louin et al., 2007; Zheng et al., 2017; Taraskina et al., 2022). In a piglet TBI model, plasma levels of glycine, ornithine, and the non-proteinogenic amino acid taurine at 24 h post-injury correlated with central injury in a regression model (Hajiaghamemar et al., 2020).

Traumatic axonal injuries (TAIs), or damage to the white matter, are a common finding in TBI, which (once established) can be detected using non-invasive neuroimaging techniques. More specifically, large haemorrhagic TAIs can be detected by computed tomography, smaller haemorrhagic TAIs by susceptibility weighted magnetic resonance imaging (MRI), and small non-haemorrhagic TAI by diffusion-weighted MR or diffusion tensor MRI (Bruggeman et al., 2021). Neurite orientation dispersion and density imaging (NODDI) is an advanced MRI modality that extends upon the principles of diffusion tensor imaging to provide greater specificity for detecting microstructural changes in the brain (Zhang et al., 2012). NODDI aims to separate the total water diffusion signal into three different and non-exchanging diffusion compartments: the isotropic free water (i.e., CSF), intra-neurite (i.e., axons and dendrites), and extra-neurite (i.e., extracellular water, neuronal cell bodies and glial cells) fractions, the proportions of which may be significantly changed in brain pathologies (Zhang et al., 2012). NODDI has already been used to examine the microstructural changes after mild TBI in humans (Churchill et al., 2017, 2019; Mayer et al., 2017; Wu et al., 2018; Palacios et al., 2020) and moderate-severe TBI in animal models (Mac Donald et al., 2007; Harris et al., 2016; To et al., 2022). Overall, NODDI detects post-TBI changes associated with neural plasticity (Harris et al., 2016; Churchill et al., 2017, 2019; Wu et al., 2018; To et al., 2022), edema (Mayer et al., 2017; Palacios et al., 2020; To et al., 2022) and axonal (Harris et al., 2016; Palacios et al., 2020) or neuronal degeneration (To et al., 2022).

Given the reported associations between plasma amino acid levels with post-TBI neuropathology and the ability of NODDI to detect microstructural changes associated with TBI pathologies, we hypothesized that TBI outcomes to NODDI might be predicted from early changes in plasma amino acid levels, conjecturally in response to alterations in the gut-brain axis and amino acid fermentation. To test this hypothesis, we undertook a prospective imaging study in rats with a standard TBI model.

## Materials and methods

### Experimental design

The experiments received approval by the Animal Research Ethics Committee (AEC) of the University of Queensland (approval number: QBI/036/16/MAIC). Fourteen Sprague–Dawley male rats (8–10 weeks old, 300–340 g) were obtained from the Animal Resource Center (ARC, Western Australia) and kept at the laboratory animal housing facility with a 12-h light-dark cycle and free access to food and water. Rats were randomly assigned to either sham surgery (*n* = 6) or TBI (*n* = 8) groups. Blood samples were drawn from the tail vein and the plasma fractions were separated and frozen for later analysis on days one, three, seven, and 60 after the surgery, whereas MRI scans, were conducted on day 60. We evaluated a plasma amino acid panel at days one, three, seven, and 60 post-surgery. Experimenters were not blinded to the animal’s experimental conditions, but personnel conducting the data processing and analysis were blinded (although TBI animals usually had obvious and gross structural changes visible on structural MRIs). All MRI data were processed semi-automatically through a processing pipeline.

### Controlled cortical impact (CCI) traumatic brain injury model

The CCI procedure was as outlined in our previous publications (Mohamed et al., 2021a; To et al., 2022). In brief, rats under isoflurane anesthesia received a 5 mm diameter craniotomy window over the right hemisphere centered at 2.5 mm posterior to bregma and 3 mm right lateral to the sagittal suture to expose the brain. A controlled cortical impact (CCI) (Osier and Dixon, 2016) was delivered to the animals in the TBI group using a pneumatically driven impactor (TBI 0310, Precision System and Instrumentation, USA) with a cylindrical 4 mm diameter tip with the following parameters: impact velocity = 5 m/s, penetration depth = 2 mm, and dwell time = 200 ms. Sham animals received the craniotomy but no impact. Overall, no animals showed conspicuous signs motor deficits after recovery from the procedure. After surgery, the wound was sutured and, following a monitored acute recovery interval, the animals were returned to their home cage. An earlier manuscript using the same injury model indicated that compared to sham animals, TBI group had slightly higher weight loss at day 1 post-surgery but this difference was no longer significant at day 3 post-injury (Mohamed et al., 2021a). No animal died after the surgery or during the course of the study, outside of planned perfusion-fixation and brain harvesting.

### Blood sample collection and inflammatory marker quantification

Tail vein venepuncture was performed at each timepoint (days one, three, seven, and 60 post-procedure) and blood was collected into 1.5 ml Eppendorf tubes containing 8 μL of 0.5 M EDTA. Additional EDTA was added to the tube to achieve a final concentration of 5 mM in the whole blood volume. Blood samples were centrifuged at 3,000 rpm at 4°C for 15 min, and the resultant plasma was filtered through glass wool by re-centrifugation at 3,000 rpm at 4°C for 15 s, and then passed through a 0.22-micron filtration column by centrifugation at 5,000 rpm at 4°C for 60 s. The filtered plasma samples were stored at −80°C for further analysis.

On the day of analysis, plasma samples were thawed to room temperature and then diluted 1:1 with 200 μM internal standard (*D,L*-norvaline, Nva; Sigma-Aldrich). The solution was deproteinated by ultrafiltration (13,800 g for 60 min at 5°C) through a membrane filter with a nominal 10 kDa molecular weight cut-off (Amicon^®^ Ultra Centrifugal Filters, Merk Millipore). Twenty microliter (20 μL) portions of filtrate were derivatized using the AccQ-Tag Ultra Derivatization Kit (Waters Corp.,) following the supplier’s recommended procedures. Standards for detection and quantitation of amino acids were prepared using the Amino Acid Standard H kit (Pierce; Thermo Fisher) with the addition of asparagine, glutamine, and tryptophan (all from Sigma-Aldrich), with Nva serving as the internal standard.

The concentrations of amino acids were determined using pre-column derivatization amino acid liquid chromatography with 6-aminoquinolyl-N-hydroxysuccinimidyl carbamate followed by separation of the derivatives and quantification by modified reversed phase ultra-performance binary gradient liquid chromatography (UPLC; Waters Corporation; Milford, MA, USA) (Cohen and Michaud, 1993; Cohen, 2000). The column employed was an ACQUITY UPLC BEH C18 1.7 μm × 100 mm column (Waters Corp.,) with detection at 260 nm (UV) and delivery of mobile phase at a flow rate of 0.7 mL/min (Cohen, 2000). This enabled a 12 min analysis time per sample.

Using the Empower software (Waters Corporation) we quantified the following amino acid concentrations: histidine, asparagine, serine, glutamine, arginine, glycine, aspartic acid, glutamic acid, threonine, alanine, proline, cystine, lysine, tyrosine, methionine, valine, isoleucine, leucine, phenylalanine, and tryptophan.

### Magnetic resonance imaging (MRI) procedure

#### Animal handling

Magnetic resonance imaging was performed on day 60 post-surgery. Anesthesia was induced using isoflurane (4% induction, 1–2% during preparation, and 0–0.3% during concurrent medetomidine infusion) in 40:60 O_2_ in medical air (2 L/min flow rate). The rats anesthetized were positioned on an MRI-compatible cradle (Bruker Biospin, Germany) with ear and tooth bars in place to reduce head motion. Rectal temperature and respiratory pattern and rates were monitored using a MR-compatible monitoring and gating system for small animals (Model 1030, Small Animal Instruments, New York, USA). After positioning the animal inside the MRI scanner, we administered the α_2_-agonist medetomidine through a peritoneal catheter as a bolus (0.05 mg/kg), immediately followed by continuous infusion (0.1 mg/kg/h). Respiration rate was in the range of 60–95 breaths per minute. Rectal temperature was maintained at 36 ± 1°C by thermostatically controlled warm water circulating in tubes embedded in the animal holding cradle (SC100, Thermo Scientific, USA).

#### MRI scans and image processing

As described in our previous publications (Mohamed et al., 2020, 2021a), MRI scans were acquired using a 9.4 T Bruker system (BioSpec 94/30USR, Bruker, Germany) and the software Paravision 6.0.1 (Bruker, Germany), along with a volume transmitter coil and a four-element array receiver coil. Anatomical imaging was performed using T2-weighted rapid-relaxation-with-enhancement (RARE) sequence with the following parameters: repetition time (TR)/Echo Time (TE) = 5900/65 ms, RARE factors = 8, number of averages = 2, FOV = 25.6 × 32 mm, matrix size = 256 × 256 × 40, and 0.5 mm-thick slices, giving an effective output spatial resolution of 0.1 × 0.125 × 0.5 mm. Diffusion-weighted images were collected using a spin-echo echo-planar imaging (EPI) sequence with TR/TE = 10000/29 ms, FOV = 24.8 × 24.8 mm, matrix size = 108 × 108 × 41, and 0.5 mm-thick slices with 0.1 mm slice gaps, giving effective output spatial resolution of 0.23 × 0.23 × 0.6 mm. Two *b*-value shells of 750, 1500 s/mm^2^, with 32 diffusion-weighted directions per shell, and 4 volumes of *b* = 0 s/mm^2^ were acquired.

Data from the MR scanner were exported in DICOM format using Paravision 6.0.1 and converted to NIFTI data format using MRIcron (Rorden and Brett, 2000). MRI images were given a modified header file with voxel size ten times larger than the original voxel size to adapt to image processing tools originally developed for human brain (Bajic et al., 2017). T2-weighted structural images were N4ITK (Tustison et al., 2010) bias field corrected [as implemented in the Advanced Normalization Tool (ANTs v.2.3.4) (Avants et al., 2014)] and skull-stripped [using 3D pulse-coupled neural networks (PCNN) (Chou et al., 2011) followed by manual editing]. Lesion-exclusion masks were created for animals in the TBI group with the lesion defined as areas with obvious hyper- or hypo-intensity and/or tissue loss on T2-weighted structural images. The pre-processed and masked structural images of the sham animals were then affine-registered to the masked SIGMA *in vivo* rat brain template (Barrière et al., 2019), using the FSL (v.6.0.4)^1^ program FLIRT (Smith et al., 2004). The sham group’s registered images were then used for an iterative non-linear image registration/template construction procedure using the Advanced Normalization Tool [ANTS v.2.3.4 (Kim et al., 2008). MultivarateTemplateConstruction2.sh] to create a study-specific sham structural template.

Image registration of structural images of all animals to the generated study-specific template was performed using the constrained cost function masking (CCFM) approach (Brett et al., 2001). This approach was implemented by registering the study-specific sham template to each animals’ pre-processed and masked structural images with an additional cost function mask that included only the “normal” parts of the brain and excluded the lesion area, using Symmetric Diffeomorphic Image Registration with Cross-Correlation (SyN-CC) (Avants et al., 2008), (implemented in ANTS). The inversion of the resulting subject-specific warping fields allows for warping of images in each subject’s structural image space to the study-specific sham template, despite gross anatomic defects in the lesioned animals.

The four *b* = 0 volumes were averaged to generate the *b* = 0 spatial representation of diffusion MRI data. Motion and eddy current corrections were performed on diffusion MRI data using FSL’s *eddy_correct* with the *b* = 0 spatial representation serving as the reference image. The representation was also N4ITK bias field corrected. Affine registration of the inhomogeneity-corrected *b* = 0 representation image to the inhomogeneity-corrected T2-weighted structural images (neither was skull-stripped) was performed as the inverse transformation was used to resample the structural brain mask (lesion-included) to the diffusion MRI space. Diffusion MRI data were fitted using neurite orientation dispersion and density imaging (NODDI) implemented in the NODDI MATLAB toolbox^2^ (Zhang et al., 2012; Tariq et al., 2016). Intra-neurite diffusion in each voxel was modeled as diffusion in zero radius cylinders, with the assumption of no lateral diffusion occurring between the neurites and a homogenous cell background; the neurite “cylinders” orientation was modeled according to Watson’s distribution and the NODDI algorithm used the tortuosity model of Szafer et al. (1995) for randomly packed cylinders. Fixed intrinsic diffusivity and fixed isotropic diffusivity were assumed to be 1.4 × 10^–9^m^2^/s and 4 × 10^–9^ m^2^/s, respectively. Neurite density index (NDI), orientation dispersion index (ODI), and isotropic diffusion volume fraction (fISO) were obtained from the diffusion model fitting.

Each rat’s inhomogeneity-corrected and masked *b* = 0 representation was registered to their own pre-processed and masked structural image (both with lesions included) using the ANTS SyN-CC registration. The warping field from this step was combined with the structural image to structural template warping field to allow for warping of the NODDI metric images (NDI, ODI, and fISO) to the study-specific sham template.

### Statistical analysis

#### Group comparisons of amino acid levels

Shapiro–Wilk normality test confirmed that plasma amino acid levels followed normal distributions. We undertook two-way analysis of variance (ANOVA) with Geisser–Greenhouse correction to examine the effect of timepoint and TBI and their interaction on the variation in the concentrations of each amino acid across all animals and measured timepoints. *Post-hoc* testing to compare the difference in each amino acid level between sham surgery and TBI groups was performed using uncorrected Fisher’s least significant difference test, with the statistical threshold set at *P* < 0.05. The aforementioned statistical analysis was performed in Prism 9 (GraphPad Inc., CA, USA).

#### Regression analysis of diffusion MRI outcomes at day 60 post-injury and TBI animals’ free amino acid panel

Data reduction for the TBI group’s plasma amino acid panel results at each timepoint was performed using principal component analysis (PCA) implemented in Prism 9. In brief, plasma levels of each amino acid across all TBI animals at each timepoint were standardized and centered so that the mean was zero and the standard deviation was one. Separate PCAs were performed for each rat’s centered and standardized amino acid levels at each time point. To minimize over-fitting, three of the principal components (PCs) were extracted from each PCA for subsequent regression analysis.

The extracted principal components values were used to perform voxel-wise multiple linear regression with the day 60 post-injury NODDI metrics as outcome variables and each TBI rat’s free amino acids panel results at the four time points as the predictor variables. Voxel-wise multiple linear regression analysis was performed by permutation inference for the general linear model (Anderson and Robinson, 2001) as implemented in FSL’s randomize (Winkler et al., 2014), with the number of permutations set to 10,000 or exhaustive, whichever was smaller. The resulting statistical maps were corrected for multiple comparisons with mass-based FSL’s threshold-free cluster enhancement (TFCE) (Smith and Nichols, 2009) and a threshold was set at *P*-value < 0.05 (two-tailed).

The voxel-wise regression analysis identified several NODDI metrics-of-interests in specific regions-of-interest (ROIs) in the brain of injured animals that could be predicted by the animals’ plasma amino acid panels early after the injury. The NODDI metrics-of-interest values of TBI animals were extracted from these ROIs, namely the NDI and fISO in the internal capsule (ic) and the fISO in the ventricles and ipsilateral impacted cortical area. Statistical map results of voxel-wise regression analysis and the ROIs of the NODDI metrics-of-interest are presented in Supplementary Figure 1. Principal component regression (PCR) was performed using Prism 9 (GraphPad Inc., CA, USA) to predict the NODDI metrics-of-interests in the ROIs from the plasma inflammatory panels of TBI animals at each timepoint. The percent variance explained for the selected PCs are shown in Table 1.

**TABLE 1:** Summary of the principle components selected from the amino acid panels at day one, three, seven, and sixty post-injury for use in the voxel-wise regression analysis with neurite orientation dispersion and density imaging (NODDI) metric maps obtained at day 60 post-injury in traumatic brain injured animals.

Day 1 post-injury amino acid levels			
PC summary	PC1	PC2	PC3
Eigenvalue	6.899	5.711	3.001
Proportion of variance	34.50%	28.56%	15.00%
Cumulative proportion of variance	34.50%	63.05%	78.06%
**Day 3 post-injury amino acid levels**			
PC summary	PC1	PC2	PC3
Eigenvalue	11.41	3.686	3.256
Proportion of variance	57.06%	18.43%	16.28%
Cumulative proportion of variance	57.06%	75.49%	91.77%
Component selection	Selected	Selected	Selected
**Day 7 post-injury amino acid levels**			
PC summary	PC1	PC2	PC3
Eigenvalue	11.1	3.39	2.64
Proportion of variance	55.69%	16.95%	13.19%
Cumulative proportion of variance	55.69%	72.64%	85.83%
Component selection	Selected	Selected	Selected
**Day 60 post-injury amino acid levels**			
PC summary	PC1	PC2	PC3
Eigenvalue	10.3	4.31	2.27
Proportion of variance	51.55%	21.55%	11.37%
Cumulative proportion of variance	51.55%	73.10%	84.47%
Component selection	Selected	Selected	Selected

#### Group comparisons of diffusion MRI metrics

Group comparisons of spatially normalized DTI, FA, NODDI metrics (NDI, ODI, and fISO) were performed using permutation inference for the general linear model (Anderson and Robinson, 2001) as implemented in FSL’s *randomize* (Winkler et al., 2014), with the number of permutations set to 10,000 or exhaustive, whichever was smaller. The resulting statistical maps were corrected for multiple comparisons with the FSL mass-based threshold-free cluster enhancement (TFCE) (Smith and Nichols, 2009) and a threshold was set at *P* < 0.05 (two-tailed).

The raw data supporting the conclusions of this article will be made available upon reasonable request to the corresponding author.

## Results

### Longitudinal changes in plasma amino acid concentrations

**FIGURE 1:**
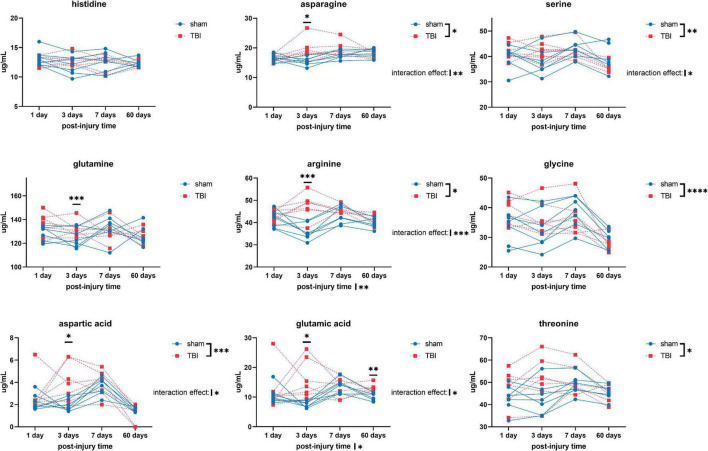
Plasma levels of amino acids: histidine, asparagine, serine, glutamine, arginine, glycine, aspartic acid, glutamic acid, and threonine of sham and traumatic brain injured (TBI) animals at day one, three, seven, and sixty post-procedure. Asterisks (*) next to sham/TBI legends, post-injury time axis label, and interaction effect indicated statistically significant group, post-injury time, and group × post-injury time effects, respectively, in a two-way repeated measures analysis of variance (ANOVA). *On the graph at each timepoint indicated significant difference between TBI and sham animals at each timepoint, Fisher’s Least Squared Difference *post-hoc* test. *P-value < 0.05, ^**^P-value < 0.01, ^***^P-value < 0.001, ^****^P-value < 0.0001.

**FIGURE 2:**
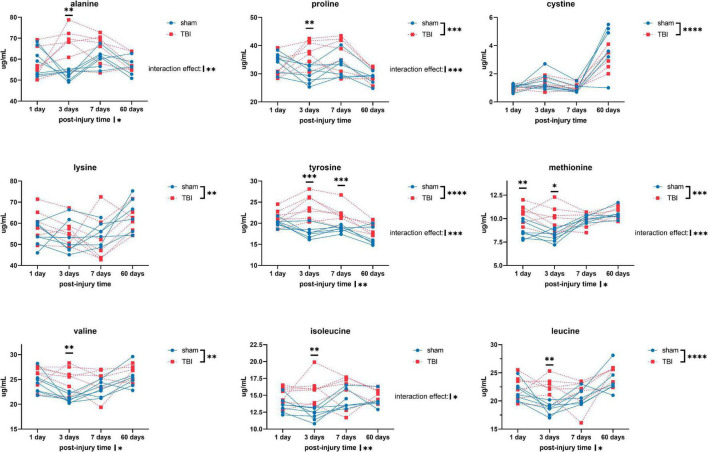
Plasma levels of amino acids: alanine, proline, cystine, lysine, tyrosine, methionine, valine, isoleucine, and leucine of sham and TBI animals at day one, three, seven, and sixty post-procedure. Asterisks (*) next to sham/TBI legends, post-injury time axis label, and interaction effect indicated statistically significant group, post-injury time, and group × post-injury time effects, respectively, in ANOVA. *On the graph at each timepoint indicated significant difference between TBI and sham animals at each timepoint, Fisher’s Least Squared Difference *post-hoc* test. *P-value < 0.05, ^**^P-value < 0.01, ^***^P-value < 0.001, ^****^P-value < 0.0001.

**FIGURE 3:**
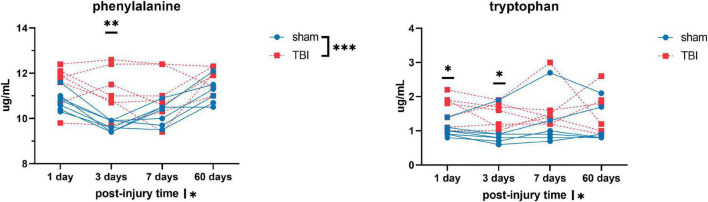
Plasma levels of amino acids: phenylalanine and tryptophan of sham and TBI animals at day one, three, seven, and sixty post-procedure. Asterisks (*) next to sham/TBI legends, post-injury time axis label, and interaction effect indicated statistically significant group, post-injury time, and group × post-injury time effects, respectively, in ANOVA. *On the graph at each timepoint indicated significant difference between TBI and sham animals at each timepoint, Fisher’s Least Squared Difference *post-hoc* test. *P-value < 0.05, ^**^P-value < 0.01, ^***^P-value < 0.001.

Overall, we detected significant effects of TBI and TBI × time effect on the plasma amino acid levels (Figures 1–3). *Post-hoc* tests showed significantly increased levels of glutamine, arginine (Figure 1), alanine, proline, the branched chain amino acids valine, isoleucine, leucine, and the aromatic amino acids tyrosine and phenylalanine (Figure 2) at days three-seven post-injury in the TBI rats.

### Correlation of plasma amino acid levels with microstructural outcomes

PCRA showed that plasma amino acid levels at days three and seven were significantly correlated with the microstructural outcomes at day 60 post-injury. Day three asparagine, serine, arginine, threonine, proline, methionine, valine, isoleucine, leucine, and phenylalanine concentrations were negatively correlated with fISO in the ventricle ROI. The regression equation was significant (*F*(3,3) = 13.15, *P*-value = 0.0312) with an adjusted R^2^ of 0.857, thus accounting for most of the variance (Table 2). Day seven glutamine, lysine (positive estimates), and aspartic acid, glutamic acid, alanine, proline, valine, isoleucine, and leucine (negative estimates) were significantly correlated with fISO in the ipsilateral cortical area. The regression equation was significant (*F*(3,3) = 21.84, *P* = 0.0154) with an adjusted R^2^ of 0.912 (Table 3). Day seven histidine, asparagine, serine, threonine, methionine, leucine, and phenylalanine levels were negatively correlated with fISO in the internal capsule ROI. The regression equation was significant (*F*(3,3) = 15.17, *P* = 0.0256) with an adjusted R^2^ of 0.876 (Table 4). Day 60 histidine, asparagine, serine, glutamine, alanine, proline, and tryptophan (positive estimates) and arginine, glutamic acid, lysine, methionine, leucine, and phenylalanine (negative estimates) were significantly correlated with NDI in the ipsilateral cortical area. The regression equation was significant (*F*(3,3) = 54.13, *P* = 0.0182) with an adjusted R^2^ of 0.976 (Table 5). Day 60 arginine, lysine, and methionine, and phenylalanine (positive estimates) and histidine, asparagine, serine, glutamine, alanine, proline, and (negative estimates) were significantly correlated with fISO in the ipsilateral cortical area. The regression equation was significant (*F*(3,2) = 20.82, *P* = 0.0462) with an adjusted R^2^ of 0.923 (Table 6).

**TABLE 2:** Result of principal component regression (PCR) predicting the isotropic diffusion fraction (fISO) quantified from the ventricle ROI from the plasma amino acid panel at day three post-injury.

Dependent variable: fISO in the ventricles ROI							
Model							
Analysis of variance	SS	DF	MS		*F*(DFn,DFd)		*P*-value
Regression	2.62E−01	3	8.74E−02		*F*(3,3) = 13.15		*P* = 0.0312
Residual	1.99E−02	3	6.65E−03				
Total	2.82E−01	6					
**Goodness of fit**							
DOF	3						
Adjusted R squared	0.857						
Day 3 plasma amino acids as variables	Estimate	95% CI (asymptotic)			|t|	*P*-value	
Intercept	2.79E + 00	1.41E + 00	to	4.17E + 00	6.444	0.0076	**
Histidine	−3.32E−03	−2.84E−02	to	2.18E−02	0.4215	0.7018	ns
Asparagine	−7.55E−03	−1.39E−02	to	−1.25E−03	3.811	0.0318	*
Serine	−6.19E−03	−9.81E−03	to	−2.56E−03	5.432	0.0122	*
Glutamine	−1.36E−03	−5.17E−03	to	2.44E−03	1.139	0.3373	ns
Arginine	−4.57E−03	−8.09E−03	to	−1.05E−03	4.128	0.0258	*
Glycine	−3.36E−03	−6.98E−03	to	2.50E−04	2.962	0.0594	ns
Aspartic acid	−2.37E−03	−1.66E−02	to	1.18E−02	0.5321	0.6316	ns
Glutamic acid	−8.13E−04	−4.57E−03	to	2.95E−03	0.6883	0.5407	ns
Threonine	−2.01E−03	−3.67E−03	to	−3.55E−04	3.865	0.0306	*
Alanine	2.70E−04	−3.24E−03	to	3.78E−03	0.2447	0.8224	ns
Proline	−2.88E−03	−5.55E−03	to	−2.15E−04	3.438	0.0413	*
Lysine	−2.55E−03	−8.82E−03	to	3.71E−03	1.298	0.2852	ns
Tyrosine	−3.20E−03	−9.03E−03	to	2.63E−03	1.746	0.1792	ns
Methionine	−1.55E−02	−2.46E−02	to	−6.42E−03	5.424	0.0123	*
Valine	−7.08E−03	−1.12E−02	to	−2.97E−03	5.482	0.0119	*
Isoleucine	−8.68E−03	−1.53E−02	to	−2.02E−03	4.147	0.0255	*
Leucine	−1.02E−02	−1.56E−02	to	−4.92E−03	6.13	0.0087	**
Phenylalanine	−1.88E−02	−3.27E−02	to	−4.93E−03	4.312	0.023	*
Tryptophan	2.61E−02	−6.19E−02	to	1.14E−01	0.9436	0.415	ns

ns, not significant, *P-value < 0.05, and **P-value < 0.01.

**TABLE 3:** Result of PCR predicting the fISO quantified from the ipsilateral cortex ROI from the plasma amino acid panel at day seven post-injury.

Dependent variable: fISO in the ipsilateral cortex ROI							
Model							
Analysis of variance	SS	DF	MS		*F*(DFn,DFd)		*P*-value
Regression	5.28E−01	3	1.76E−01		*F*(3,3) = 21.84		*P* = 0.0154
Residual	2.42E−02	3	8.06E−03				
Total	5.52E−01	6					
**Goodness of fit**							
DOF	3						
Adjusted R squared	0.912						
Day 7 plasma amino acids as variables	Estimate	95% CI (asymptotic)			|t|	*P*-value	
Intercept	9.99E−01	−1.31E + 00	to	3.31E + 00	1.375	0.2627	ns
Histidine	2.30E−03	−1.10E−02	to	1.56E−02	0.5505	0.6203	ns
Asparagine	−4.28E−04	−1.02E−02	to	9.36E−03	0.1392	0.8981	ns
Serine	−2.70E−03	−8.88E−03	to	3.48E−03	1.39	0.2586	ns
Glutamine	4.80E−03	7.63E−04	to	8.85E−03	3.783	0.0324	*
Arginine	6.08E−03	−1.29E−02	to	2.50E−02	1.021	0.3824	ns
Glycine	−1.67E−03	−6.55E−03	to	3.21E−03	1.087	0.3564	ns
Aspartic acid	−2.10E−02	−3.78E−02	to	−4.18E−03	3.973	0.0285	*
Glutamic acid	−7.91E−03	−1.42E−02	to	−1.65E−03	4.02	0.0276	*
Threonine	−1.59E−03	−5.28E−03	to	2.11E−03	1.365	0.2655	ns
Alanine	−5.30E−03	−8.12E−03	to	−2.48E−03	5.987	0.0093	**
Proline	−5.49E−03	−7.97E−03	to	−3.02E−03	7.069	0.0058	**
Lysine	3.45E−03	1.39E−03	to	5.50E−03	5.343	0.0128	*
Tyrosine	−6.62E−03	−1.89E−02	to	5.70E−03	1.71	0.1859	ns
Methionine	1.28E−02	−2.45E−02	to	5.01E−02	1.093	0.3544	ns
Valine	−1.26E−02	−1.93E−02	to	−5.80E−03	5.911	0.0097	**
Isoleucine	−1.37E−02	−1.96E−02	to	−7.85E−03	7.418	0.0051	**
Leucine	−1.11E−02	−1.56E−02	to	−6.55E−03	7.772	0.0044	**
Phenylalanine	−8.56E−03	−2.97E−02	to	1.26E−02	1.288	0.2882	ns
Tryptophan	−1.69E−02	−6.86E−02	to	3.49E−02	1.037	0.3758	ns

ns, not significant, *P-value < 0.05, and **P-value < 0.01.

**TABLE 4:** Result of PCR predicting the fISO quantified from the internal capsule ROI from the plasma amino acid panel at day seven post-injury.

Dependent variable: fISO in the internal capsule ROI							
Model							
Analysis of variance	SS	DF	MS		*F*(DFn,DFd)		*P*-value
Regression	1.11E−03	3	3.75E−04		*F*(3,3) = 15.17		*P* = 0.0256
Residual	9.26E−05	3	2.47E−05				
Total	1.20E−03	6					
**Goodness of fit**							
DOF	3						
Adjusted R squared	0.876						
Day 7 plasma amino acids as variables	Estimate	95% CI (asymptotic)			|t|	*P*-value	
Intercept	2.76E−01	1.48E−01	to	4.04E−01	6.86	0.0063	**
Histidine	−7.52E−04	−1.49E−03	to	−1.65E−05	3.25	0.0474	*
Asparagine	−7.54E−04	−1.30E−03	to	−2.12E−04	4.42	0.0214	*
Serine	−4.62E−04	−8.04E−04	to	−1.20E−04	4.3	0.0232	*
Glutamine	−1.07E−04	−3.31E−04	to	1.17E−04	1.53	0.2246	ns
Arginine	−1.04E−03	−2.09E−03	to	8.22E−06	3.16	0.051	ns
Glycine	−1.69E−04	−4.39E−04	to	1.02E−04	1.99	0.1413	ns
Aspartic acid	−6.36E−04	−1.57E−03	to	2.95E−04	2.17	0.1182	ns
Glutamic acid	−2.07E−04	−5.54E−04	to	1.39E−04	1.9	0.1531	ns
Threonine	−2.36E−04	−4.40E−04	to	−3.08E−05	3.66	0.0352	*
Alanine	−4.07E−05	−1.97E−04	to	1.15E−04	0.831	0.4671	ns
Proline	−1.13E−04	−2.50E−04	to	2.36E−05	2.63	0.078	ns
Lysine	9.29E−06	−1.04E−04	to	1.23E−04	0.26	0.8118	ns
Tyrosine	−4.69E−04	−1.15E−03	to	2.14E−04	2.19	0.1167	ns
Methionine	−2.29E−03	−4.35E−03	to	−2.18E−04	3.52	0.039	*
Valine	−1.89E−04	−5.64E−04	to	1.85E−04	1.61	0.2063	ns
Isoleucine	−3.13E−04	−6.40E−04	to	1.37E−05	3.05	0.0555	ns
Leucine	−3.41E−04	−5.93E−04	to	−9.00E−05	4.32	0.0228	*
Phenylalanine	−1.69E−03	−2.86E−03	to	−5.16E−04	4.58	0.0195	*
Tryptophan	−7.66E−04	−3.63E−03	to	2.10E−03	0.851	0.4575	ns

ns, not significant, *P-value < 0.05, and **P-value < 0.01.

**TABLE 5:** Result of PCR predicting the neurite dispersion index (NDI) quantified from the ipsilateral cortex ROI from the plasma amino acid panel at day sixty post-injury.

Dependent variable: NDI in the ipsilateral cortex ROI							
Model							
Analysis of variance	SS	DF	MS		*F*(DFn,DFd)		*P*-value
Regression	1.06E−01	3	3.53E−02		*F*(3,3) = 54.13		*P* = 0.0182
Residual	1.30E−03	3	6.52E−04				
Total	1.07E−01	6					
**Goodness of fit**							
DOF	3						
Adjusted R squared	0.976						
Day 60 plasma amino acids as variables	Estimate	95% CI (asymptotic)			|t|	*P*−value	
Intercept	7.90E−01	−2.54E−01	to	1.83E + 00	3.257	0.0827	ns
Histidine	4.06E−02	2.64E−02	to	5.47E−02	12.3	0.0065	**
Asparagine	6.11E−03	1.47E−03	to	1.07E−02	5.663	0.0298	*
Serine	1.06E−02	6.23E−03	to	1.50E−02	10.42	0.0091	**
Glutamine	4.60E−03	2.84E−03	to	6.37E−03	11.22	0.0078	**
Arginine	−2.03E−02	−2.77E−02	to	−1.28E−02	11.68	0.0073	**
Glycine	1.54E−03	−2.49E−03	to	5.57E−03	1.641	0.2424	ns
Aspartic acid	9.83E−04	−7.70E−03	to	9.67E−03	0.4867	0.6746	ns
Glutamic acid	−5.86E−03	−9.32E−03	to	−2.39E−03	7.275	0.0184	*
Threonine	−6.18E−04	−2.56E−03	to	1.32E−03	1.373	0.3036	ns
Alanine	3.79E−03	2.29E−03	to	5.30E−03	10.87	0.0084	**
Proline	5.36E−03	2.89E−03	to	7.84E−03	9.314	0.0113	*
Lysine	−7.29E−03	−9.81E−03	to	−4.78E−03	12.47	0.0064	**
Tyrosine	8.19E−04	−6.20E−03	to	7.84E−03	0.5017	0.6657	ns
Methionine	−2.51E−02	−4.25E−02	to	−7.57E−03	6.166	0.0253	*
Valine	−9.41E−04	−4.91E−03	to	3.03E−03	1.02	0.4151	ns
Isoleucine	1.80E−03	−4.58E−03	to	8.18E−03	1.212	0.3492	ns
Leucine	−7.11E−03	−1.26E−02	to	−1.61E−03	5.567	0.0308	*
Phenylalanine	−5.13E−02	−7.43E−02	to	−2.83E−02	9.582	0.0107	*
Tryptophan	2.70E−02	1.33E−02	to	4.08E−02	8.452	0.0137	*

ns, not significant, *P-value < 0.05, and **P-value < 0.01.

**TABLE 6:** Result of PCR predicting the fISO quantified from the ipsilateral cortex ROI from the plasma amino acid panel at day sixty post-injury.

Dependent variable: fISO in the ipsilateral cortex ROI							
Model							
Analysis of variance	SS	DF	MS		*F*(DFn,DFd)		*P*-value
Regression	4.92E−01	3	1.64E−01		*F*(3,2) = 20.82		*P* = 0.0462
Residual	1.58E−02	3	7.88E−03				
Total	5.08E−01	6					
**Goodness of fit**							
DOF	3						
Adjusted R squared	0.923						
Day 60 plasma amino acids as variables	Estimate	95% CI (asymptotic)			|t|	*P*-value	
Intercept	−5.07E−01	−4.14E + 00	to	3.12E + 00	0.6009	0.6089	ns
Histidine	−8.95E−02	−1.39E−01	to	−4.01E−02	7.802	0.016	*
Asparagine	−1.93E−02	−3.54E−02	to	−3.12E−03	5.136	0.0359	*
Serine	−1.97E−02	−3.49E−02	to	−4.48E−03	5.569	0.0308	*
Glutamine	−8.81E−03	−1.50E−02	to	−2.67E−03	6.173	0.0253	*
Arginine	4.27E−02	1.68E−02	to	6.87E−02	7.083	0.0194	*
Glycine	2.10E−03	−1.19E−02	to	1.61E−02	0.6439	0.5856	ns
Aspartic acid	−1.56E−02	−4.58E−02	to	1.46E−02	2.218	0.1568	ns
Glutamic acid	1.15E−02	−5.32E−04	to	2.36E−02	4.113	0.0543	ns
Threonine	2.65E−03	−4.09E−03	to	9.39E−03	1.692	0.2328	ns
Alanine	−8.55E−03	−1.38E−02	to	−3.33E−03	7.045	0.0196	*
Proline	−1.38E−02	−2.24E−02	to	−5.18E−03	6.89	0.0204	*
Lysine	1.57E−02	6.97E−03	to	2.45E−02	7.729	0.0163	*
Tyrosine	5.37E−03	−1.91E−02	to	2.98E−02	0.9455	0.4442	ns
Methionine	6.25E−02	1.63E−03	to	1.23E−01	4.418	0.0476	*
Valine	−4.08E−03	−1.79E−02	to	9.74E−03	1.269	0.3321	ns
Isoleucine	−1.35E−02	−3.57E−02	to	8.72E−03	2.613	0.1206	ns
Leucine	7.18E−03	−1.19E−02	to	2.63E−02	1.616	0.2475	ns
Phenylalanine	1.08E−01	2.81E−02	to	1.88E−01	5.814	0.0283	*
Tryptophan	−4.05E−02	−8.83E−02	to	7.41E−03	3.637	0.068	ns

ns, not significant, *P-value < 0.05.

Results of group comparison of registered NODDI metrics of this cohort has been reported previously (To et al., 2022). Of relevance to the correlations in this study, decreased NDI was observed in the ipsilateral cortical area and increased fISO in the ventricles and impacted cortical area.

## Discussion

In this study we have shown that elevated plasma concentrations of amino acids, specifically asparagine, serine, arginine, proline, valine, leucine, isoleucine, and phenylalanine, at day three post TBI were predictive of lower fISO measures in the ventricles at day 60 post-injury. The lower fISO measures were specifically indicative of lesser ventriculomegaly pathology specifically, and possibly of lesser TBI pathology in general. While we cannot draw causal inferences, we argue that the elevated availability of these amino acids mediated neuroprotective effects in the TBI injury model.

Neurite orientation dispersion and density imaging separates the total water diffusion signal into three non-exchanging diffusion compartments, one of which is the isotropic diffusion fraction, which describes the fraction of the total diffusion signal in a given voxel that is attributable to isotropic diffusion characteristic of free water, i.e., the cerebrospinal fluid in the ventricles (Zhang et al., 2012). The increase fISO in the ventricles occurred in areas with significant hyperintensities on T2-weighted images and ventricular volume (To et al., 2022), where increased fISO in the ventricles is an indication of edema and ventricular enlargement. In our study, the sham animals, or lesioned animals with relatively less ventriculomegaly or smaller ventricles had greater partial volume effects. Hence, the surrounding non-ventricular tissue contributed more non-ventricular diffusion signals to the ventricular voxels, so that the apparent fISO in the ventricles appear to be lower. In enlarged ventricles or oedemic lesions with strong T2-weighted hyperintensities, there were lesser partial volume effects, and the free water in the cerebral spinal fluid consequently contributed more to the total diffusion signal, such that the apparent fISO value approached the “pure” isotropic diffusion fraction. Thus, the increased apparent fISO signal is properly attributable to the enlargement of the ventricles (in ventricular voxels) and/or edema (in voxels that otherwise will be intact gray matter in the shams). Ventriculomegaly and hydrocephalus are common findings in animal (Dixon et al., 1999; Zhao et al., 2014; Mohamed et al., 2021a) and human TBIs (Edna and Cappelen, 1987; Gale et al., 1995; Anderson et al., 1996; Poca et al., 2005; Mohamed et al., 2021b).

### Amino acids in traumatic brain injury

In the rat group with the controlled cortical impact model, we saw significantly increased plasma levels of, most notably, arginine, alanine, proline, valine, isoleucine, leucine, and phenylalanine during days 3–7 days post-injury. These increases had normalized at day 60 post-injury, although there remained certain correlations with structural markers. There have been conflicting results from previous studies on this topic; some studies reported subacute “nitrogen loss” (Flakoll et al., 1995), namely a general decrease in the plasma levels of the majority of the amino acids in humans (Flakoll et al., 1995; Petersen et al., 1996; Yi et al., 2016) and in animal models of TBI (Louin et al., 2007; Zheng et al., 2017; Taraskina et al., 2022). Among the specific amino acids undergoing a decline in previous studies were alanine (Deutschman, 1987), arginine (Flakoll et al., 1995; Petersen et al., 1996), glutamine (Deutschman, 1987; Flakoll et al., 1995; Petersen et al., 1996; Yi et al., 2016), proline (Flakoll et al., 1995; Louin et al., 2007; Zheng et al., 2017), serine (Flakoll et al., 1995; Yi et al., 2016; Hajiaghamemar et al., 2020), taurine (Flakoll et al., 1995; Hajiaghamemar et al., 2020), threonine (Flakoll et al., 1995; Zheng et al., 2017), tryptophan (Flakoll et al., 1995; Taraskina et al., 2022), and the branched-chain amino acids (BAA) leucine, isoleucine, and valine (Vuille-Dit-Bille et al., 2012; Jeter et al., 2013). On the other hand, a small number of other studies have also shown a mixed pattern of increased and decreased plasma amino acids, both in human TBI (Deutschman, 1987; Flakoll et al., 1995; Vuille-Dit-Bille et al., 2012) and animal models (Hajiaghamemar et al., 2020). The amino acids most consistently found to be elevated in these studies were the BAAs and phenylalanine (Deutschman, 1987; Flakoll et al., 1995), which is consistent with our present results. On the other hand, others have also found the opposite trends (Vuille-Dit-Bille et al., 2012; Jeter et al., 2013). Administration of BAA supplements post-TBI appeared to exert beneficial and neuroprotective effects (Aquilani et al., 2008; Cole et al., 2010) [for review, see Sharma et al., 2018]. Thus, the general increase of plasma amino acids levels, especially BAA and phenylalanine, appear to have been a beneficial part of the recovery process. To resolve the contradiction between general trends of nitrogen loss observed in human clinical studies (Deutschman, 1987; Flakoll et al., 1995; Petersen et al., 1996; Vuille-Dit-Bille et al., 2012; Jeter et al., 2013; Yi et al., 2016) and the overall increase in plasma amino acid levels post-injury seen in this and other studies, we invoke the differences in overall survival and well-being of human TBI patients versus model animals. Moderate-to-severe clinical TBIs often require intensive care (Stocker, 2019) including those seen in prior studies of post-TBI plasma amino acids: the participants were hospitalized even in cases of mild TBI (Jeter et al., 2013) and required ventilation (Flakoll et al., 1995) or more often intensive care unit treatment (Deutschman, 1987; Petersen et al., 1996; Vuille-Dit-Bille et al., 2011, 2012). One study reported a 9% mortality in the TBI group (Petersen et al., 1996). In contrast, our CCI procedure entails a standardized and circumscribed percussion injury in free-breathing anesthetized animals, the recovery process required no mechanical ventilation, and no animal died from the injury, as is typical in our hands. Nonetheless the CCI model of TBI here utilized impact to moderate-severe severity and resulted in significant and obvious brain tissue loss visible on MRI (To et al., 2022). We suppose that this rat TBI model, despite the ostensibly moderate-severe injury severity, does not translate to the life-threating severity of the brain injuries in patients recruited in clinical studies of amino acid concentrations.

These observations call for some speculation about the mechanism whereby TBI might influence plasma amino acid levels. There is an abundance of evidence that TBI has effects on inflammatory pathways and the gut brain axis (Ferrara et al., 2022). In particular, TBI profoundly altered the gut microbiome, and that transfer of fecal microbiota can rescue some of the behavioral and brain structural effects of TBI (Davis et al., 2022). Post-TBI changes in gut microbiota composition were associated with alterations in the plasma levels of citrulline (an arginine metabolite) and tryptophan (Taraskina et al., 2022). A plausible causal mechanism for such effects involves dysautonomia and systemic inflammation after TBI, which propagate to gastrointestinal changes such as dysmotility and increased mucosal permeability (Hanscom et al., 2021). One of the limitations of the current study is the absence of formal analysis of the rats’ microbiota post-TBI, such that our proposed mechanism remains a speculation, albeit one that had support in prior literature. Our interpretation is that the observed changes in the plasma levels of BAAs and other amino acids reflect altered amino acid fermentation in the gut. As such, the alterations evident in the early days after rat TBI may be surrogates for the severity of the injury or may conversely confer protection from structural injury as manifest in the NODDI-MR findings at day 60 after the injury.

### Effects of sub-acute post-TBI changes in amino acids on long-term microstructural outcomes

As previously discussed, BAA supplementation has had neuroprotective effects post-TBI in animal and human trials. The benefits of elevated BAA levels were further evidenced by present results showing higher plasma levels of BAAs at day three post-injury among TBI animals, occurring in association with lower fISO in the ventricles and less severe hydrocephalus and ventriculomegaly at day 60 post-injury to NODDI-MR examination. Day seven branched-chain amino acid levels, although not significantly different in the TBI cohort compared to shams, also negatively correlated with fISO measures in the ipsilateral cortical area, meaning that the TBI animals with higher levels of branched-chain amino acids suffered less edema or tissue loss in the impacted cortical area. While this may be further evidence for the neuroprotective role of BAAs, we note that higher BAA plasma levels among human TBI patients were associated with poorer clinical indicators, namely higher intracranial pressure and lower cerebral oxygen consumption (Vuille-Dit-Bille et al., 2012).

Present findings of day three-seven post injury elevations of certain plasma amino acids proved to be associated with less severe long-term microstructure outcomes, notably for cases of serine, arginine, asparagine, and phenylalanine, which are also known for their neuroprotective roles. Serine reduces neuroexcitotoxicity, regulates microglia polarization, decreases inflammation, improves cerebral blood flow and promotes survival, proliferation, and differentiation of neural stem cells (Ye et al., 2021). Arginine exerts a neuroprotective effect by suppression of the hypoxia inducible factor 1α (HIF-1α)/lactate dehydrogenase (LDHA)-mediated inflammatory response in the microglia (Chen et al., 2020). Asparagine, while being a non-essential amino acid, must be synthesized locally in the brain by asparagine synthase *via* the ATP-dependent conversion of aspartate and glutamate (Ruzzo et al., 2013; Sprute et al., 2019), indicating its importance in brain development and potentially in post-injury repair/recovery. Higher plasma levels of phenylalanine were associated with lower intracranial pressure and increased cerebral oxygen consumption post-TBI (Vuille-Dit-Bille et al., 2012).

In conclusion, we found in this study a generalized increase in the plasma levels of amino acids post-injury in a rat model of moderate-severe open-head TBI. The effects were most pronounced for amino acids with known neuroprotective roles, i.e., serine, and arginine, and asparagine, and the BAAs leucine, isoleucine, and valine. Injured animals with higher plasma levels of these amino acids at days three-seven post injury had lesser TBI pathologies at 60 days follow-up, namely less severe ventriculomegaly and less edema or tissue loss in the ipsilateral cortical area. Present results also highlighted a potentially important difference or limitation of animal TBI models in relation to clinical TBI: rats and rodents may have inherently more robust or adaptive brain injury repair and recovery as compared to humans faced with clinically significant injury.

## Data availability statement

The raw data supporting the conclusions of this article will be made available upon request to the corresponding author, without undue reservation.

## Ethics statement

The animal study was reviewed and approved by the Animal Research Ethics Committee (AEC) of the University of Queensland (approval number: QBI/036/16/MAIC).

## Author contributions

XVT conducted the imaging data processing and overall data analysis, wrote the first draft, and made the figures and tables. AM designed the experiments and conducted the animal experiments and imaging. PC revised the subsequent drafts of the manuscript and interpreted the results. FN devised the first conception and design of the study, acquired funding, revised the manuscript, and provided overall supervision of the study. All authors wrote sections of the manuscript, contributed to the manuscript revision, read, and approved the submitted version.

## Footnotes

1
https://fsl.fmrib.ox.ac.uk/fsl/fslwiki

2
https://www.nitrc.org/projects/noddi_toolbox

## Supplementary material

Supplementary Figure 1 Results of principal component regression analyses predicting **(A)** neurite density index (NDI) and **(B)** isotropic diffusion fraction (fISO) from the first independent component (IC) of the amino acid panel at day three post-injury and **(C)** NDI from the first IC of the amino acid panel at day seven post-injury. **(D)** Spatial maps of the regions-of-interest (ROIs) used for quantification of NODDI metrics from the internal capsule (red), ipsilateral cortex (green), and ventricles (blue).
