# Supplementary figures and images for: Association of sub-acute changes in plasma amino acid levels with long-term brain pathologies in a rat model of moderate-severe traumatic brain injury
Source: Front Neurosci. 2023 Jan 6;16:1014081. doi: 10.3389/fnins.2022.1014081 (PMC9853432; doi:10.3389/fnins.2022.1014081)

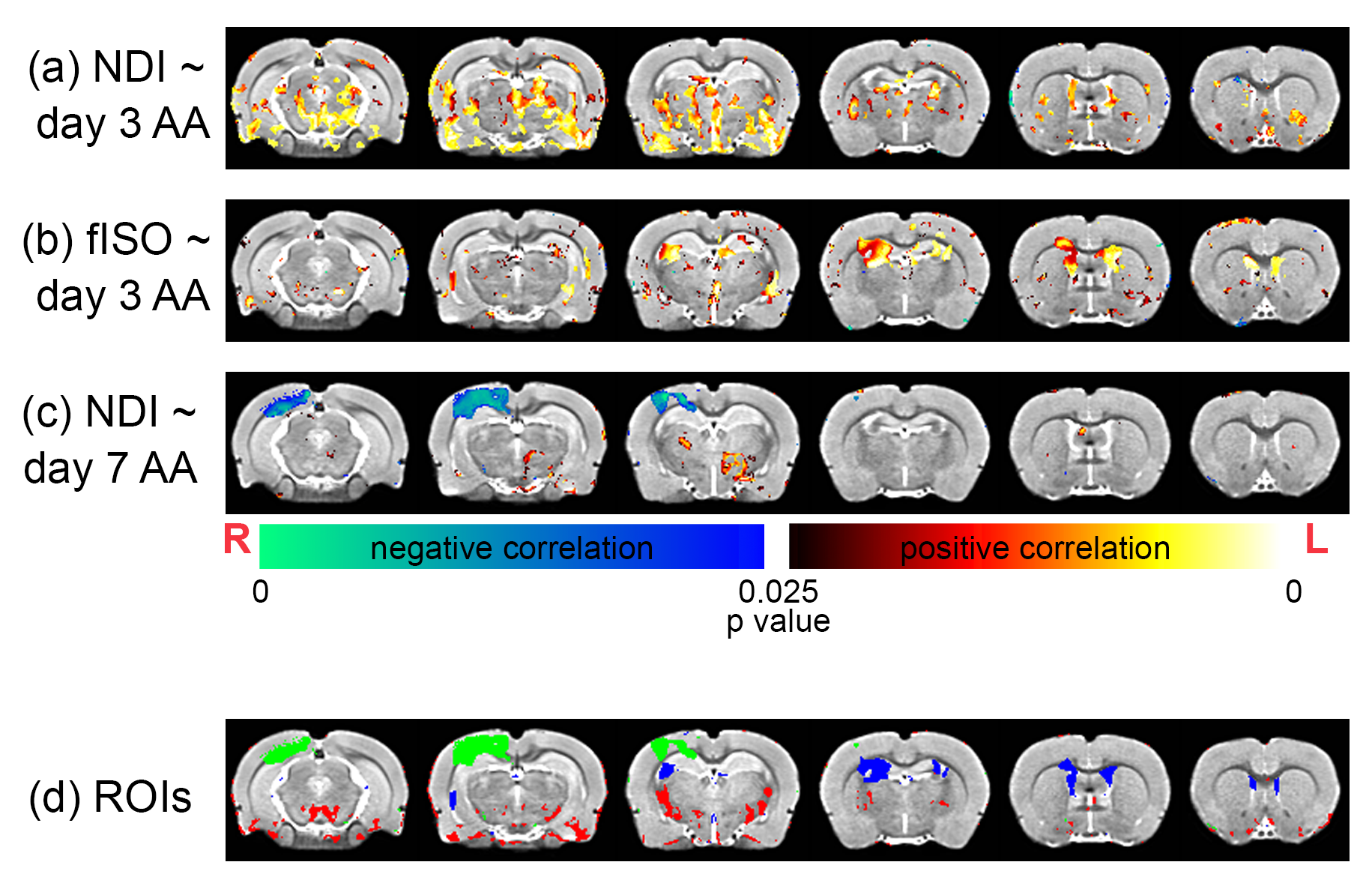

Supplement: Supplementary Figure 1 — Results of principal component regression analyses predicting (A) neurite density index (NDI) and (B) isotropic diffusion fraction (fISO) from the first independent component (IC) of the amino acid panel at day three post-injury and (C) NDI from the first IC of the amino acid panel at day seven post-injury. (D) Spatial maps of the regions-of-interest (ROIs) used for quantification of NODDI metrics from the internal capsule (red), ipsilateral cortex (green), and ventricles (blue). [file Image_1.TIF]
